# Improved sensitivity, safety and laboratory turnaround time in the diagnosis of pulmonary tuberculosis by use of bleach sedimentation

**DOI:** 10.4102/ajlm.v4i1.117

**Published:** 2015-11-18

**Authors:** Ameh James, Kingsley Ochei, Nnamdi Emenyonu, Lovett Lawson

**Affiliations:** 1 Family Health International 360, Abuja, Nigeria; 2 Keystone Laboratories International, Diagnostic Unit, Abuja, Nigeria; 3 Zankli Medical Centre, Plot 1021, B5, Shehu Yaradua Way, Abuja, Nigeria

## Abstract

**Background:**

Inadequate diagnostic processes and human resources in laboratories contribute to a high burden of tuberculosis (TB) in low- and middle-income countries. Direct smear microscopy is relied on for TB diagnosis; however, sensitivity rates vary. To improve sensitivity of direct microscopy, the researchers employed several approaches, including sputum digestion and concentration of acid-fast bacilli (AFB), a technique which uses commercial bleach.

**Objectives:**

This study compared methods used to diagnose active *Mycobacterium tuberculosis* infections.

**Methods:**

Three sputum specimens were collected from each of 340 participants in Abuja, Nigeria, over two consecutive days. Direct microscopy was performed on all specimens; following microscopy, one specimen from each patient was selected randomly for bleach sedimentation and one for Lowenstein-Jensen culture.

**Results:**

Direct microscopy produced 28.8% AFB-positive results, whilst bleach sedimentation resulted in 30.3%. When compared with the cultures, 26.5% were AFB true positive using direct microscopy and 27.1% using bleach sedimentation. Whilst the specificity rate between these two methods was not statistically significant (*P* = 0.548), the sensitivity rate was significant (*P* = 0.004).

**Conclusion:**

Based on these results, bleach increases the sensitivity of microscopy compared with direct smear and has similar specificity. When diagnosing new cases of pulmonary TB, one bleach-digested smear is as sensitive as three direct smears, reducing waiting times for patients and ensuring the safety of laboratory technicians.

## Introduction

Pulmonary tuberculosis (TB) has a large impact on developing country populations, especially in sub-Saharan Africa where its burden has been increased by the rapid spread of human immunodeficiency virus (HIV). HIV infection impairs cell-mediated immunity, which provides an opportunity for the reactivation of TB, making individuals living with HIV more susceptible to this pathogen. Furthermore, HIV can reduce the sputum positivity rate, leading to false sputum-smear-negative TB.^1,2,3^ TB can be caused by any member of the *Mycobacterium tuberculosis* complex (*M. tuberculosis, M. bovis, M. africanum, M. caprae, M. microti, M. cannettii* and *M. pinnipedi*). The factors responsible for the high burden of TB in low- and middle-income countries (LMIC) include poor diagnostic processes and inadequate human resources in laboratories. The most widely-available diagnostic test in these settings is direct smear microscopy (hereafter called direct microscopy), which is used for TB diagnosis. This test is cheap, simple and highly specific for *Mycobacterium tuberculosis*.^4^

In 1995, through the Directly Observed Therapy Short (DOTS) course strategy, the World Health Organization (WHO) set a global target for 2005 to detect 70% of new smear-positive cases.^5^ Unfortunately, this target has not been met, in part due to inaccurate diagnoses.^6^ Several investigators reported varying sensitivity rates of direct microscopy, ranging from 20% – 60% in some settings to 80% in another setting.^7,8,9,10,11,12,13, 14,15^ This finding has led to the search for alternative techniques to improve the sensitivity rate of direct microscopy, resulting in the development of several methods to optimise the procedure. Amongst these approaches is the sputum digestion and concentration of acid-fast bacilli (AFB) using commercial bleach (sodium hypochlorite), a widely-available household commodity, instead of the standard sodium hydroxide (NaOH) concentration method. The technique has been shown to improve the clarity of the smears and increase the yield of bacilli for easy detection.^16^ To achieve these benefits, investigators used centrifugation and sedimentation concentration methods. Bleach centrifugation and sedimentation studies have been widely reported and reviewed by different authors to determine the suitability of the method for TB diagnosis in LMIC.^6,17,18^

Only a few reports that used Lowenstein-Jensen (LJ) culture are available and none of the studies were conducted in Nigeria.^19,20^ The only published study in Nigeria utilised the BACTEC MGIT 960 (Beckton Dickinson, Franklin Lakes, New Jersey, United States), but because of a lack of required technology the researchers could not differentiate between the species of *M. tuberculosis* complex.^21^ Furthermore, most published studies compared either bleach centrifugation or sedimentation with direct microscopy rather than with the gold standard of mycobacterial culture.^13,22,23,24^ Additionally, in a recent review by Cattamanchi et al., lack of validation of these studies has been challenged.^18^ As a result, this study was conducted to compare the method of bleach sedimentation for less than one hour with direct microscopy and LJ culture. This study reports the sensitivity, specificity and positive and negative predictive values of bleach sedimentation and direct microscopy as compared to LJ culture, the reference standard.

## Methods

### Settings and patient recruitment

The DOTS clinics in six different government-owned and managed health facilities across the Federal Capital Territory (FCT) in Abuja, Nigeria, referred 340 patients to the Zankli Medical Centre. The referring health facilities included Maitama District Hospital, Asokoro District Hospital, Wuse General Hospital, Gwagwalada Specialist Hospital, Kubwa General Hospital and Gwarimpa General Hospital. The participants, 192 men and 148 women aged between 10 and 64 years, were prospectively enrolled in the study between November 2004 and July 2005. The participants referred from the six sites were assessed for suspected pulmonary TB. Participants who did not submit three specimens over a two-day period and participants receiving anti-TB treatment were excluded from the study.

### Sample collection

Each of the 340 participants submitted three sputum specimens over two consecutive days. In total, 1020 specimens were collected. The first specimen was collected during the patients’ first visit to Zankli Medical Centre, whilst the second was collected by the patients at their homes. Patients were given instructions on how to collect an appropriate specimen for diagnosis of pulmonary TB; this process included taking the sample early in the morning before brushing the teeth. The third specimen was taken at the Zankli Medical Centre when patients delivered their second specimen. The two specimens taken at the Zankli Medical Centre were produced by patients in an open and well-ventilated area of the facility. Laboratory technicians performed direct microscopy on all specimens collected and randomly selected one specimen for bleach sedimentation and one specimen for LJ culturing. All diagnostic tests gave conclusive results for the 340 participants.

### Direct microscopy

Smears (1 cm × 2 cm) were made from the purulent sputum, air-dried and heat-fixed on a hot plate at 85 °C for two to three minutes, then stained by the Ziehl-Neelsen (ZN) method (1% filtered carbol-fuchsin and 0.1% malachite green or methylene blue).

### Bleach sedimentation

An equal volume of undiluted commercial bleach (5% sodium hypochlorite) was added to the remaining part of the specimen. The specimen cup was tightly closed and the contents were vigorously shaken by hand for about 20 seconds; the cup was then placed at an angle of 45 ° and remained, undisturbed, at room temperature (18 °C – 30 °C) for 30 minutes. The sediment was gently withdrawn using a disposable Pasteur pipette and a drop of the deposit was transferred to a slide. This was used to make a smear of approximately 1 cm × 2 cm. The smear was air-dried, heat-fixed and stained using the ZN method.

### Microscopic examination and interpretation

Both the direct and bleach smears were read using the oil immersion lens (×100) of an ordinary light microscope by experienced microscopists who were blinded to the results. Slides were read again in the case of discordant results. For both direct and bleach slides, positive and negative smears were defined according to the National Tuberculosis and Leprosy Control Program's AFB grading system (Table 1).^7^ A patient was reported smear-positive for TB if at least one to nine AFB were seen in 100 high-power fields. Hence, the study reported on the number of patients diagnosed with active *M. tuberculosis* infections.

**TABLE 1:** Guide to acid-fast bacilli (AFB) microscopy interpretation according to the National Tuberculosis and Leprosy Control Program's AFB grading system.^7^

Number of AFB	Recording/reporting
No AFB in 100 fields	Negative
1–9 AFB seen in 100 fields	Actual number
1–9 AFB in 10 fields	+
1–9 AFB per field	++
> 10 AFB per field	+++

### Sputum decontamination (modified Petroff method), culture and isolation of *M. tuberculosis*

Sputum for the LJ culture technique was selected randomly from the participants’ three specimens. An equal volume of 4% sodium hydroxide was added to 5 mL of sputum in a 30 mL screw-cap tube. This tube was capped tightly and shaken to digest the sputum; thereafter, the tube stood at room temperature for 15 minutes with occasional shaking. The mixture was centrifuged at 3000 revolutions per minute for 15 minutes. The supernatant was carefully decanted, after which the deposit was resuspended with 15 mL of sterile normal saline and re-centrifuged at the same rate. The supernatant was removed and the tube sediment of the second centrifugation was inoculated on an LJ agar slope and incubated at 37 °C ± 2 °C. For the first three days, the specimen was observed daily for signs of possible contamination. At weekly intervals over the following six to 10 weeks, the culture was examined regularly for the isolation of *M. tuberculosis*. Positive and negative growth controls were always included, using wild strains of *M. tuberculosis* complex and sterile distilled water, respectively. The isolates were identified as *Mycobacterium* species by the nitrate reduction test, catalase heat-labile test and ZN smear method.

### Statistical analyses

The proportions (sensitivity, specificity and negative and positive predictive values) were calculated using standard definitions.^25^ The estimated proportions were then compared using the two-sample test of proportions for large samples (using the normal approximation to the binomial distribution) and estimating the confidence intervals in the process. In particular, an immediate form of the two-sample test of proportions was applied using the prtesti command in Stata software version 11 (STATA Corp LP, College Station, Texas, United States). A *P*-value of < 0.05 was considered statistically significant.

### Ethical considerations

Verbal informed consent was obtained from the participants and ethical approval granted by the ethical committee of the FCT Hospital Management Board and the Zankli Medical Centre.

## Results

**FIGURE 1:**
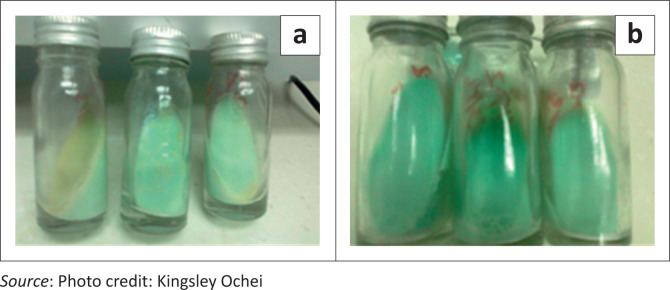
Positive (a) and negative (b) Lowenstein-Jensen cultures for *M. tuberculosis*.

Of the 340 participants evaluated for TB, 28.8% were AFB-positive using direct smear and 30.3% using bleach sedimentation (Table 2). When compared with mycobacterial culture (Figure 1), the gold standard, 26.5% of samples were true positive for AFB using direct smear and 27.1% using bleach sedimentation (Table 3). This comparison determined the sensitivity and specificity rates of the two methods (Table 4). Whilst the difference in the specificity rate between the two evaluated methods was not significantly significant (*P* = 0.548), the difference between sensitivity rates was significant (*P* = 0.004), indicating that bleach sedimentation is more sensitive. Furthermore, unlike the difference between positive predictive values (*P* = 0.542), the difference in the negative predictive values was statistically significant (*P* = 0.003), demonstrating that the bleach sedimentation method more accurately identified the AFB-negative participants who were not infected with *M. tuberculosis*, in contrast to those with false-negative results.

**TABLE 2:** Results of Ziehl-Neelsen microscopy for acid-fast bacilli using both direct and bleach sedimentation-treated sputa, Abuja, Nigeria, November 2004 to July 2005.

Result	Direct smear (three sputa), *n*(%)	Bleach sedimentation (one sputum), *n*(%)
Positive	98 (28.8)	103 (30.3)
Negative	242 (71.2)	237 (69.7)
**Total**	**340 (100.0)**	**340 (100.0)**

Note: Total number of participants = 340 (men = 192, women = 148, age range = 10 to 64 years).

**TABLE 3:** Comparison of Ziehl-Neelsen microscopy for acid-fast bacilli using direct and bleach sedimentation-treated sputa with culture on Lowenstein-Jensen media following sodium hydroxide decontamination, Abuja, Nigeria, November 2004 to July 2005.

Result	NaOH + LJ culture, *n*(%)	Direct smear (three sputa) microscopy, *n*(%)	Bleach sedimentation (one sputum) microscopy, *n*(%)
True positive	106 (31.2)	90 (26.5)	92 (27.1)
True negative	234 (68.8)	226 (66.5)	234 (68.8)
False positive	0 (0.0)	8 (2.3)	11 (3.2)
False negative	0 (0.0)	16 (4.7)	3 (0.9)

NaOH, sodium hydroxide; LJ, Lowenstein-Jensen.

**TABLE 4:** Diagnostic accuracy of direct microscopy and bleach sedimentation-treated sputa compared with Lowenstein-Jensen culture following sodium hydroxide decontamination, Abuja, Nigeria, November 2004 to July 2005.

Diagnostic accuracy	Direct microscopy		Bleach sedimentation		Differences†	*P*-value‡
	*n*/*N*	% (95%Cl)	*n*/*N*	% (95%Cl)	% (95%Cl)	
Sensitivity	90/106	84.9 (78.1–91.7)	92/95	96.8 (93.3–100.4)	−11.9 (−19.6–[−4.3])	0.004
Specificity	226/234	96.6 (94.3–98.9)	234/245	95.5 (92.9–98.1)	1.1 (−2.4–4.6)	0.548
PPV	90/98	91.8 (86.4–97.3)	92/103	89.3 (83.4–95.3)	2.5 (−5.5–10.6)	0.542
NPV	226/242	93.4 (90.3–96.5)	234/237	98.7 (97.3–100.2)	−5.3 (−8.8–1.9)	0.003

PPV, positive predictive value; NPV, negative predictive value; CI, confidence interval.

†, A two-sample test of proportions was used to determine the percentage differences and 95% CI. The applied test of proportions was based on the prtesti command in the Stata statistical package.

‡, A *P*-value of < 0.05 was considered statistically significant.

## Discussion

In this study, researchers found that bleach increased the sensitivity of microscopy compared with the direct smear and had similar specificity. This finding supports the outcomes reported in other studies that have evaluated bleach sedimentation whilst using culture as a reference standard.^10,19,20^

Despite its drawbacks, direct microscopy remains the cornerstone of TB diagnosis in developing countries, particularly in sub-Saharan Africa. Direct microscopy has low sensitivity, as described in a review of 14 studies.^6^ However, in this study, direct microscopy showed a relative increase in sensitivity. This finding cannot be extrapolated; however, as it was likely the result of the setting in which this study was conducted: a research laboratory with greater time resources than routine diagnostic laboratories, particularly government-owned or public health facilities.

Though not a problem in this study, an additional drawback of direct microscopy without bleach sedimentation is that it requires the submission of three specimens, which can result in increased dropout rates. Many patients are unable to afford the cost of travelling to a health centre to submit one or multiple samples.^26^ Therefore, the failure to attain the WHO's global target of 70% case detection is not surprising, given that the burden of TB is in developing countries where direct microscopy is still routinely used for diagnosis.

The use of bleach sedimentation, requiring only one specimen, has several benefits, such as greatly reducing the workload of overextended laboratories in resource-poor settings and reducing the long turnaround time associated with both direct microscopy and the overnight sedimentation method employed in other studies.^10,19^ Additional advantages associated with bleach sedimentation are safety, ease of manipulation and cost-effectiveness.^21^ Significantly, bleach is readily available even in remote parts of the developing world, whereas sodium hydroxide, traditionally used in laboratories, may be difficult or even impossible to acquire. One study showed that the use of 3% bleach for 20 minutes completely sterilises sputum containing *M. tuberculosis*.^27^ However, a previous study found total sterilisation in only 93.6% of AFB-positive sputa after treatment with 5% bleach for between 15 minutes and three hours.^28^ Regardless, the use of household-strength bleach in processing sputa for AFB microscopy has significant laboratory safety advantages, as it sterilises the majority of processed sputa, reducing technicians’ risk of exposure to AFB.

### Limitations

This study was conducted in a controlled laboratory, unlike a typical public health laboratory where laboratorians are expected to meet a particular turnaround time. Thus, this study was carefully carried out without any ‘time pressure’. It is suggested that a similar study should be performed in a typical routine diagnostic laboratory.

### Recommendations

This study recommends the use of bleach for sputum microscopy, as it helps to increase the sensitivity of the test and reduces the work load on the laboratory. It also offers protection for the laboratory personnel's against the bacteria.

### Conclusion

In conclusion, in settings with a high TB burden, bleach sedimentation may improve sensitivity and laboratory safety in the diagnosis of *M. tuberculosis* and reduce waiting periods for test results. Evidence shows that one bleach-digested sputum smear may be more sensitive than three direct smears in the detection of new cases of pulmonary TB. This diagnostic test could increase the detection rate of new cases of TB in rural areas in developing countries.
